# Building public trust and acceptance towards spray-on RNAi biopesticides: lessons from current ethical, legal and social discourses

**DOI:** 10.1080/21645698.2025.2510735

**Published:** 2025-05-27

**Authors:** Ariyani Rinaldi, Nurzatil Sharleeza Mat Jalaluddin, Rosila Bee Mohd Hussain, Adilah Abdul Ghapor

**Affiliations:** a Institute for Advanced Studies, Advanced Studies Complex, Universiti Malaya, Kuala Lumpur, Malaysia; b Department of Science and Technology Studies, Faculty of Science, Universiti Malaya, Kuala Lumpur, Malaysia; c Centre for Research in Biotechnology for Agriculture (CEBAR), Universiti Malaya, Kuala Lumpur, Malaysia; d Department of Anthropology and Sociology, Faculty of Arts And Social Sciences, Universiti Malaya, Kuala Lumpur, Malaysia; e Department of Decision Science, Faculty of Business and Economics, Universiti Malaya, Kuala Lumpur, Malaysia

**Keywords:** ELSI, responsible research innovation, governance, Trust, non-transformative RNAI

## Abstract

Advances in New Plant Breeding Techniques (NBTs), particularly spray-on RNA interference (RNAi) biopesticides, necessitates a reevaluation of existing regulatory and governance frameworks. While spray-on RNAi technologies offer promising solutions for sustainable crop protection and targeted pest control without altering plant genomes, they also raise important ethical, legal, and social implications (ELSI). This paper explores current ELSI discourses surrounding spray-on RNAi biopesticides, such as issues of environmental risk, regulatory ambiguity, corporate control and public acceptance. The study also highlights the importance to incorporate trust as an ethical element in developing regulatory and governance framework for the RNAi technology to increase public acceptance toward the technology. These findings contribute to the broader discourse on the governance of novel biotechnologies in agriculture, offering guidance for future regulatory design tailored to the unique characteristics of spray-on RNAi-based interventions.

## 1. Introduction

Understanding on how RNA molecules regulate gene expression has revolutionized molecular biology and genetic engineering tools and applications. One such tool is using RNA interference (RNAi), which is a natural regulatory mechanism that can be used to silence target genes in target organisms. The gene silencing effect can be induced by sequences-specific double-stranded RNAs (dsRNAs), which when being detected by the cells, the RNAs will be used as a guiding template and will be directed to specific site on-target RNAs that then cleaves the complementary RNA sequences. RNA degradation ultimately inhibits protein synthesis.^1–3^

In recent years, the RNAi strategy has been exploited using a non-transformative approach, which does not require insertion of a foreign gene to produce silencing RNA molecules in a target organism. Contrary to the traditional genetic modification (GM) approach, spray-induced gene silencing (SIGS) effects can be achieved by topically applying active dsRNAs directly onto the target organism to elicit silencing effects and downregulate gene expression. These naked dsRNAs can be produced using *in vitro* and *in vivo* methods, such as microbe- and yeast-mediated expression systems.^4^

Since the first RNAi silencing observation, known as co-suppression, in the transgenic *Petunia hybrida* L,^5^ numerous research works have been carried out to develop sprayable RNAi-biopesticide for crop protection (reviewed in.^6,7^ However, these naked dsRNAs are liable to nuclease degradation when being exposed to UV radiation, in-plant environment, water and soil environment.^8^ Therefore, active RNA molecules are often capsulated in nanocarriers to prolong its stability and activity when being applied onto target crops.^9^ To our best knowledge, several spray-on RNAi biopesticides have been trialed in the field, such as patent-protected BioClay,^10^ BioDirect,^8^ Calantha,^11^ RNAi insecticide targeting Colorado Potato Beetle *Mesh* gene,^12^ and yeast-based RNAi pesticide developed by Renaissance BioScience.^13^ These field trials were conducted in Australia, the United States, Canada and Europe. However, only one foliar RNA biopesticide has been succesfully registered for crop protection, which is Calantha^TM^, a commercial product developed by GreenLight Biosciences that received approval from the U.S. Environmental Protection Agency (EPA).^14^ Calantha^TM^ contains active ingredient ledprona, which is a dsRNA insecticide designed to selectively control the Colorado potato beetle (CPB), *Leptinotarsa decemlineata* that can destroy potato crops and reduce tuber production.^15^

Proponents of spray-on RNAi biopesticide have narrated that RNAi technology is a revolutionary tool capable of enhancing crop productivity, improving farm cost efficiency while minimizing health and environmental hazards.^16^ Recent discussions highlighted that RNAi-based pesticides not only offer targeted pest control with reduced ecological risk but also align with the European Union’s Farm-to-Fork strategy, which aims to reduce dependency on conventional agrochemicals while maintaining agricultural productivity and food security.^17^ RNAi technology is viewed as complementary to integrated pest management^16,18^ and precision agriculture, supporting key targets under the European Commission’s Green Deal and Biodiversity Strategy 2030, such as reduced pesticide residues, improved soil health, and lower greenhouse gas emissions.^17^

However, critics have been framing sprayable RNAi-based pesticides in open fields as a threat to the environment, citing that the open-air experiment will pose serious risks to exposed organisms.^19^ The RNAi debate has also been surrounded with the uncertainty around the heritability of gene-silencing effects, which often being portrayed as transient and non-heritable.^20^ Arguably, the transient gene silencing effect may not hold true for certain organisms or grafted plants and therefore, raises an important regulatory question of what constitutes “transient gene silencing.”^21^

The rise of sprayable RNAi-based pesticide introduces additional regulatory ambiguity and governance challenges to policymakers.^22^ While RNAi technology offers precision and efficiency compared to traditional GMOs, their rapid development and uncertain long-term effects necessitate robust risk assessment frameworks. The initial discourses surrounding sprayable RNAi-based pesticides centered around the necessity to understand environmental risks such as off-target effects and implications from persistence and spread of dsRNA molecules in the environment.^23^ Additionally, other issues such as human health risk assessments and management,^24^ intellectual property rights,^25^ public acceptance,^26,27^ food labels^28^ and farmer dependency^19^ are equally important aspects for consideration when drafting a regulatory and governance framework for sprayable RNAi-based pesticides. Therefore, this paper seeks to comprehensively review critical themes and debates surrounding ELSI discourses of RNAi technology, discuss the importance of addressing trust issues toward emerging biotechnology, as well as identify research priorities and policy gaps that require attention to ensure the regulatory and governance framework for New Plant Breeding (NBT) techniques, especially spray-on RNAi-based biopesticides, is responsible and inclusive.

## 2. ELSI Discourses Surrounding Sprayable RNAi-Based Pesticides

The development and deployment of sprayable RNAi-based pesticides raise a number of ethical, legal, and social implications (ELSI) that extend beyond the technical considerations of efficacy and delivery. These concerns mirror and expand upon those associated with genetically modified (GM) organisms, while also presenting novel challenges specific to the unique nature of the non-transformative RNAi technology.

From an ethical perspective, one of the primary concerns is the potential for off-target effects, whereby double-stranded RNA (dsRNA) may unintentionally silence genes in non-target organisms, including beneficial insects, soil microbiota, or even humans upon exposure through environmental contact.^29,30^ The potential for such unintended consequences poses dilemmas around ecological responsibility, which as a principle of environmental ethics, recognizes the natural world, including both biotic and abiotic components, as a fragile and interdependent system entrusted to human stewardship.^31,32^ It extends beyond human-centered concerns to emphasize the intrinsic value of ecosystems and the necessity of maintaining natural equilibrium for the continuity of life.^33^

As a targeted pest control method, RNAi technologies are often promoted as more ecologically friendly than conventional chemical pesticides due to its naturalness and reduced toxicity.^13^ However, ecological responsibility entails more than reducing toxicity as it requires acknowledging the interconnectedness of living and non-living systems and ensuring that pest control interventions do not undermine ecological balance.^31^ This includes addressing potential off-target effects on non-target organisms, such as pollinators, soil fauna, or aquatic species, whose ecological functions are critical to long-term agricultural sustainability.^29^ Much of the criticism was attributed to the importance of preserving pollinator populations and plant–pollinator interactions, which if lost, can disrupt ecological equilibrium leading to altered food web dynamics. Beyond their ecological function, pollinators contribute substantially to agricultural productivity, economic stability and global food security.^34^

In light of these complexities and uncertainties, adopting a precautionary approach to risk management becomes ethically justified. The precautionary approach from the ecological ethics perspective, emphasizes the anticipation and mitigation of uncertain and potential irreversible harms associated with emerging technologies to an ecosystem. In the case of RNAi-based pesticides, there are still knowledge gaps about unintentional gene silencing effects of non-target organisms on various species including honey bees,^35^ slender springtails^36^ and monarch butterflies.^37^ Thus, by conducting thorough scientific investigation and stringent ecological risk assessments, especially evaluating potential impacts on pollinators prior to large-scale application, is desirable.^20^ However, it is also important to note that taking this step may lead to an overestimation of potential impacts and an underestimation of the effectiveness of mitigation measures.^38^ It is therefore important to strike a balance between mitigating potential negative impacts and addressing potentially devastating impacts to food security if the technology is not moved forward.

In addition, ethical debates also arise around the corporate ownership and accessibility of RNAi technologies, as cost-effectiveness of the technology can be an issue for smallholder farmers, thereby exacerbating existing inequalities in agricultural innovation.^19^ Corporate control via patenting regime^36^ and broad patent claims leading to challenges to navigate “patent thickets” of RNA-mediated gene suppression technologies have been discussed for transgenic RNAi crops, as well as for gene-edited crops.^39–42^ Previous patent landscape studies on RNAi-based pesticides illustrated that private companies including large agricultural firms such as Bayer CropScience that merged with Monsanto and Syngenta who acquired Belgian firm Devgen hold multiple patent portfolios on sprayable RNAi-based pesticide.^43^ The patent claims covered several aspects such as composition of matter which is the actual dsRNA sequences, dsRNA delivery systems such as nanoparticle formulations and clay nanosheets, methods of application for example foliar sprays and seed coating, as well as target genes or pathogens.^43^ The patenting trend for the RNAi technology is positive as observed by the rising number of patents that were filed by the private companies in developing and developed countries since the last two decades, which further illustrated the increased corporate patenting activities in this space.^43^ Currently, the cost of producing dsRNA using in vitro methods is reported as minimum as 0.50 cents/gram with an estimate of 2–10 g needed for each hectare of farm.^44^ With this figure, there are still doubts whether smallholder farmers especially in developing countries will have an inclusive access to the technology.^45^

**Figure 1.**
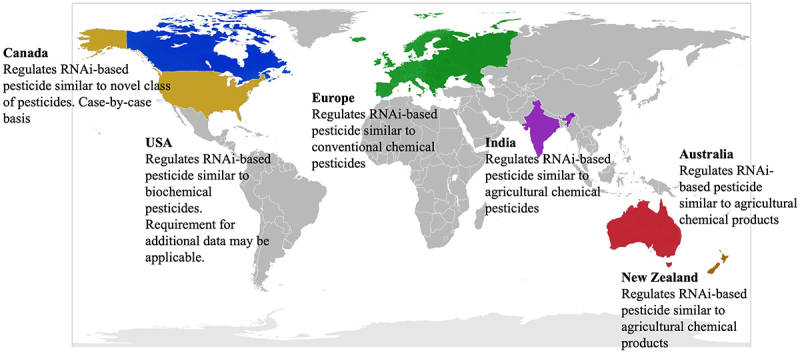
Global status of regulations for RNAi-based pesticides.

From the legal point of view, the spray-on RNAi biopesticide presents regulatory classification and harmonization challenges.^13,22^ Proponents advocated for regulatory ambiguities to be clarified and appropriate and standardized science-based risk assessment to be adopted for evaluation of sprayable RNAi-based pesticides.^13^ Some have argued that plant protection products (PPP) risk assessment should be adapted to evaluate unique risks associated with spray-on dsRNA-based pesticides^46^ while others insisted for the products to be regulated as another form of genetic engineering.^19^ Several countries such as the US, Canada, Europe, New Zealand and Australia regulate RNAi-based pesticides under existing chemical pesticides regulations (See Figure 1 and Table 1). However, many other countries have yet to clarify their regulatory approaches for the technology.^22,44^

**Table 1.** Regulatory landscape for spray-on RNAi-based biopesticides (adapted from)^8,13^.

Jurisdiction	Regulatory Authority	Act and Regulation	Data requirements
Australia	The Australian Pesticides and Veterinary Medicines Authority	Agricultural and Veterinary Chemicals Code Act 1994	Similar to agricultural chemical products, such as risk and environmental fate of the active ingredient and assessment of the formulation (including nanocarriers)
USA	US Environmental Protection Agency	Pesticide regulation 40 CFR 158	Similar to biochemical pesticides, such as technical grade of active ingredient tested in mammalia and non-target organism, product acute toxicity studies and exposure, and additional data for externally applied product
Canada	Pest Management Regulatory Agency	Pest Control Products Act 2002	Similar to novel class of pesticides and data requirements are on a case-by-case basis
Europe	The European Food Safety Authority	Regulation (EC) 1107/2009	Similar to conventional chemical pesticides, involving assessment of the active compound and the plant protection product containing the active compound
New Zealand	The Environmental Protection Agency	Hazardous Substances and New Organisms Act 1996	Similar to agricultural chemical product
India	Genetic Engineering Appraisal Committee	India Insecticides Act 1968	Similar to agricultural chemical pesticides

Previous reports have indicated mixed public views on the development and use of sprayable RNAi-based pesticides. Opponents of the technology have argued that the scientific process to produce RNAi-based pesticides use genetic engineering techniques and thus raising unique risks and concerns. They have also highlighted that the assumption of these RNAi-based products as “eco-friendly” and “natural” lacks sufficient scientific justification.^19^ This is because synthetic interfering RNAs, which are being developed for insecticidal purposes, may vary significantly in their biological effects depending on their specific sequences and the organisms to which they are applied.^47^ Moreover, these RNAs are modified to be encapsulated nanomaterials to improve their stability and efficacy, which may give rise to unique risks that are yet fully understood.^47^

Proponents however sought to distinguish sprayable RNAi-based pesticides from traditional GMOs, suggesting that the RNAi pesticide does not integrate foreign genetic material into the plant genome upon spraying.^26^ From a public acceptance perspective, this argument plays a significant role, as one of the important ethical and religious objections to genetically modified organisms (GMOs) stems from the perception that they are “unnatural,” particularly due to the integration of genetic material from multiple species onto the crop genome.^48^ In addition to efforts to distinguish RNAi-based pesticides from earlier criticisms associated with genetically modified (GM) agriculture, proponents argue that RNAi technologies offer significant economic benefits for the European Union and are essential to addressing future global food security challenges.^17^

These ELSI concerns highlighted the challenges in garnering positive public perception and acceptance toward the use of RNAi technology for biopesticide sprays in agriculture. Learning from the previous experience with GMOs, public views toward biotechnology can be diverse, depending on cultural norms, personal values, ethics and societal taboos.^49,50^ As such, the extent that public accepted biotechnology has been associated with their trust in technological developers or those responsible for risk assessments of the technology who share the same values to ensure public safety.^27,51^ As sprayable RNAi-based biopesticides advance, building public trust and incorporating trust as an ethical value in technological governance are essential for the innovation to be accepted by the society and more fully integrated into agricultural practices. The following sections review several trust models that have been used to evaluate public acceptance and discuss appropriate regulatory and governance models that incorporate trust in the framework.

## 3. Different Theories and Dimensions to Analyse Trust

In a “post-truth” era, where the society regularly scrutinizes scientific and technological claims, fostering trust becomes essential to facilitate transparent communication and meaningful public engagement on sprayable RNAi-based pesticides.^44,52^ As such, building trust in the government’s ability to regulate risk and in the ethical research conduct and competence of technological developers can enhance social acceptance of the RNAi technology.^53^ Conversely, public trust erodes when there are perceived conflicts of interests between stakeholders and failure to acknowledge scientific knowledge limits that ultimately affect public acceptance.^54^

The development of trust models in biotechnology has evolved significantly to address the multifaceted concerns surrounding products arising from genetic modification (GM) and new plant breeding techniques (NBTs). Initially, trust was viewed through the perspective of technical risk assessments, with early models operating under the assumption that public skepticism could be mitigated by increasing scientific knowledge.^55,56^ However, as it became evident that public concerns were not solely about scientific risk but also about ethical, social, and cultural values, more sophisticated trust models were introduced.^57,58^

One of the foundational models, the trust, confidence and cooperation (TCC) model, was proposed by Earle and Siegrist.^59^ The TCC model provides a framework to describe the differences between trust and confidence and explain that trust and confidence as interacting sources of cooperation.^59^ Within this framework, trust is referred as shared values, and can be characterized by morality, integrity, caring, benevolence, inferred traits and intentions while confidence is associated with past performance and encompasses multiple aspects of ability.^60^ Hence, the basis of trust is the trusted individual is expected to behave as a trustworthy person would in a similar situation.^60^ The TCC model has been applied to evaluate the acceptance of Swiss citizens toward GM field experiments, which were carried out after a moratorium on gene technology in 2005.^60^ The study showed that public acceptance of GM field experiments was significantly influenced by three factors, firstly is trust due to shared values in economy/health and environment, secondly is trust due to shared values in honesty of industry and scientists and thirdly is competence (confidence).^60^ Additionally, the study showed that moral conviction, outcome fairness, and procedural fairness were significant predictors of GM acceptance, with procedural fairness having a stronger influence on individuals with high moral convictions.^60^

To further explore the dynamics between trust and risk perception, the causal chain model was introduced. The causal chain model proposes that trust influences risk perceptions and risk perceptions influence the activity or technological acceptance^61,62^ while the associationist view interprets trust as a consequence of technological acceptance, or trust as an indicator of a more general attitude toward a technology or activity.^63^ These two models have been compared to evaluate relationships between trust and acceptance toward GM food. Findings in the past studies have shown that the relationship between trust, perceived risk and attitudes toward food technologies are better supported by the associationist view than the causal chain model.^63,64^

Building on the associationist view, the Integrative Model has been proposed to reconcile the analysis of dimensions of trust, the salient value similarity and the associationist view of trust.^65,66^ The salient value similarity approach proposes that trust is shaped by perceived shared values, or the extent to which individuals believe others or organizations view situations similarly to them.^65,66^ According to the Integrative Model, attitudes influence value similarity and value similarity influences general trust. This, in turn, impacts the level of trust in the regulation of risks, such as those associated with GM foods, ultimately affecting their acceptability.^65,66^

An alternative model to evaluate trust was the SPARTA (S – subjective norms, P – perceived behavioral control, A- attitudes, R – risk perception, T – trust and A – “alia”/all other variables) model that integrates risk perceptions and trust in the theory of planned behavior.^67^ The multi-attribute model was employed to elucidate the links among attitudes and beliefs, purchase intentions, trust, and risk perceptions, leading to the acceptance of GM foods.^67,68^ Along this vein, previous studies have argued that attitudes toward GM technology and trust in institutions have an influence on perceptions of risks and benefits of GM products, while socio-economic characteristics of consumers have an effect in the decision-making process regarding the intention to purchase or avoid GM foods.^67,68^

Trust has been contextualized as a predictor for public acceptance toward biotechnological products.^57^ Biotechnology, like many other emerging technologies, offer numerous potential benefits but at the same time present large uncertainties and expectations about the real and perceived risks that the technology may have on society.^69^ Most laypeople do not have intricate knowledge of benefits and risks of biotechnology. In the early days when biotechnology was introduced to the mainstream, majority of the general population do not understand the basics about gene technology and not even being able to differentiate between DNA, molecules and radiation.^70,71^ Adding to this knowledge gap, the society wonders if the innovation process reflects their values and interests and becomes concerned about possible harms arising from decisions made by the technology developers, users, and regulators.^58,72^ Since the society has limited knowledge and understanding about biotechnology, they are unable to evaluate the accuracy and reliability of complex information about risks and benefits associated with the technology. In this circumstance, lay judgments and decisions rely on social trust to reduce uncertainties when making decisions and judgments on risks and benefits of a biotechnological product.^58^

Social trust refers to the perception that social actors or institutions such as regulators who share the same values as the lay people, being honest and credible, and have the capability to manage a particular technology.^58,73^ Findings from early surveys suggested that variation in public attitudes toward GM food is related with social trust in trusted institutions such as regulators, scientists, universities and industry, rather than absolute levels of trust such as culture-specific and individual differences in knowledge of science.^74^ In the context of public trust in social actors, trust is defined as a combination of trust factors on competence, transparency, public interest, and honesty.^75^ Based on the ratings, public voted for evaluators such as scientists as the most trusted entity, watchdogs such as environmental organizations were moderately trusted, and industry and government were least trusted.^75^ In addition, Gutteling et al. observed a trend where the government was the least trusted entity in matters concerning genetically modified (GM) food, whereas non-governmental organizations (NGOs) garnered more trust than other stakeholders.^76^ This disparity in trust correlated with attitudes and participation in activities such as demonstrations and signing petitions against GM initiatives. Specifically, their findings suggested that a diminished trust in governmental institutions or an enhanced trust in NGOs was associated with more negative attitudes toward GM foods and a reduced level of acceptance compared to those with an opposite trust distribution, which the public has more faith in government and less in NGOs.^76^ Table 2 summarizes selected research on trust-related food biotechnology literature in the past twenty years.

**Table 2.** Selected literature on public trust in social actors relevant for GM food.

No	Context	Types of Trust Measured	Measurement of trust	Data collection method	Data analysis method	Key Findings	Recommendations for building trust	Reference
1.	Relationship between trust, perceptions of risks and benefits and gene technology acceptance	Trust in scientists and researchers, food, pharmaceutical, and agricultural companies.	5-point Likert scale	Telephone interviews in Switzerland. (*N* = 693)	Structural equation modeling	Females indicated more trust. Trust has an indirect impact on biotechnological acceptance	More knowledge about risk perception and acceptance of gene technology is needed.	^58^
2.	Role of social trust and knowledge in the perception of hazards	Trust in managing authorities	7-point Likert scale	A survey at Western Washington University. (*N* = 91)	Correlation analysis	Public depends on social trust when assessing risks without personal knowledge	Recognize the varying sources of trust depending on the technology.	^69^
3.	Trust as a factor for acceptance of GM food	Trust in government	Likert scale	Telephone survey between March and April 2001 in the United States. (*N* = 342)	Ordered Probit model	Individuals who trust government are more likely to accept GM foods than those who lack of trust	Trust in government is crucial for the acceptance of GM food	^77^
4.	Trust in institutions with roles and resources related to GM food	Competence, transparency, public interest, and honesty	5-point Likert scale	Computer-assisted telephone interview in the United States. (*N* = 409)	Exploratory factor analysis	Evaluators (whom are scientists, universities, medical professionals) are the most trusted.	Build trust through open dialogue and broader information consideration.	^75^
5.	Trust in governance, government and NGOs as important predictors for public acceptance of GM food in the Netherlands	Trust in governance, government and NGOs	5-point Likert scale	Telephone interviews in June 2001 in the Netherlands. (*N* = 1,019)	Factor analysis, multivariate analyses, hierarchical regression analysis	Trust in governance is an important constraint for GM food development	Involve the public in decision-making, consider consumer interests, and address individual perceptions of biotechnology’s impact.	^76^
6.	Hazard-related trust and risk perception	Competence, care and values	5-point Likert scale	Online survey in November 2002 in the UK. (*N*=1,142)	Confirmatory factor analysis	Shared values as the important factor to explain risk perception.	Risk perception can attenuated by persuading scientists and risk managers are trustworthy and the risks can be dealt with.	^78^
7.	Trust in government agencies, farmers, scientists and corporations	Competence and confidence	5-point Likert scale	Telephone survey from March to May 2004 in the US Southwestern (*N* = 432)	Principal factor analysis	Trust in institutions is mediated through perceived benefits	Building public trust by conveying benefits to consumers	^79^
8.	Trust in organisations and experts, and evaluated by five trust dimensions	Honesty, shared values, prediction, knowledge, importance	5-point Likert scale	Mail survey in May and June 2004 in the United States (*N* = 363)	Exploratory factor analysis, regression analysis	Respondents regarded as activities, and industry groups as trustworthy; Perceived honesty is important to predict trust across groups	Understanding trust elements across the groups	^80^
9.	Trust in organisations in explaining attitudes towards GM products	Trust in scientists, regulators and watchdogs.	10-point Likert scale	Computer-assisted telephone interview from 2003 to 2012. (*N* = 8821)	Structural equation modeling	Public trust in scientists and watchdogs is a strong predictor of attitudes toward GM plants for food.	Prioritise building trust in scientists and regulating organisations related to GM products	^74^
10.	Public attitudes toward science and technology and their trust in governing organisations regulating GM	Trust in governance, GM actors, and GM regulations,	5-point Likert scale	Online surveys and telephone interviews from March and April 2015 in the Netherlands. (*N* = 1,208)	Regression analysis	The Dutch show positivity towards GM, trust in regulations, and optimism about GM governance.	Positive attitude towards science and technology link to more trust in government and business.	^81^
11.	Effects of epistemic trust and social trust on public acceptance of GM food	Epistemic trust and social trust (trust in public and industrial organisations)	7-point Likert scale	Survey in China. (*N* = 1,091)	Partial least squares structural equation modeling (PLS-SEM)	Epistemic trust is an important antecedent of perceived benefits and risks; Trust in industrial entities negatively impacts perceived risks; Trust in public entities positively impacts perceived benefits.	Public entities must properly execute their responsibilities to regulate risk management of GM food; industrial entities must improve the transparency for risk control information to enhance goodwill trust.	^82^
12	Linkage between gene-edited food crop adoption is linked and public trust in institutions and values	Institutional trust	5-point Likert scale	Survey in United States. (*N* = 1,980)	Ordinal regression models	GEF doption hinges on public trust in institutions that oversee GEF development, especially university scientists.	Public trust in GEFs and labels can be garnered through oversight by universities, advocacy groups, and government food regulators.	^83^
13.	Cultivating trust: Public perception of RNAi technologies in agriculture	Trust in science (scientific institutions and scientific processes)	10-point Likert scale	Online survey in Italy (*N* = 709)	Partial least squares structural equation modeling (PLS-SEM)	The main element lowering public opposition to RNAi technologies in agriculture is trust in science, not scientific knowledge.	Transparent communication, address emotional and societal concerns, and regulatory transparency.	^84^
14	Beekeepers Support the Use of RNA Interference (RNAi) to Control Varroa destructor	Institutional trust	5-point Likert scale	Mixed-method (survey *N* = 175 and FGD with 13 participants)	Descriptive statistics, Spearman correlation and Q methodology (PCA)	Most beekeepers agree that RNAi is a safer option than pesticides.	Scientific transparency, public education, clear distinction from GM, and inclusive communication strategies.	^27^

## 4. Trust and Biotechnology Governance

Assessments on biotechnological applications such as transgenic RNAi have been risk-based, that focused on technical evaluation of risks to environmental and human health, favoring technical experts in the process.^85^ Since transgenic RNAi is regulated using existing GMO framework,^46^ the inclusion of non-technical experts tends to occur in the later stage of risk assessment, with public participation is limited to providing invited comments on assessments defined by experts and have limited influence on decision-making process.^86^ As biotechnology been “problematised” from different aspects, not only on risk, but also on ethical, social and economic concerns, the scopes of governance options have broadened to incorporate wider knowledge sources from a range of actors and stakeholders.^87^ These actors and stakeholders, such as environmental groups, consumer organizations, commissioned scientific expert panels or ethics committees, participate in different forms of engagements and offer different scopes of policy advices in response to the risk, ethical, social and economic arguments.^54,87^ These engagements can be carried out using methods of consensus conferences, dialogs, citizen juries and panels and science shops.

The emergence of controversies further led to increasing calls for public stakeholder participation from the early “upstream” stage of technological development before nearing toward commercialization when key decisions are made, financial and non-financial resources are heavily invested, and trajectories have been set.^88,89^ The involvement of lay public in debates received many criticisms, because deliberations and decisions render complex decision-making processes and the lay perspective may have incompatibility with the dominant views of respective risk, ethical and economic arguments.^89^ Nonetheless, “upstream” engagement provides learning opportunities for technological developers including researchers to learn from when participating in public dialogs.^13^ An example is the “FORTiGe – Forschungsverbund Tiergesundheit durch Genomik” project that conducted multiple public stakeholder engagements to explore technical and social feasibility of using CRISPR-Cas9-based gene editing for Bavarian livestock agriculture.^90^ While the project was deemed successful as the co-design of editing targets and proof-of-concept experiments were successfully achieved, the multi-stakeholder consultations however indicated that it would be unlikely that the gene-editing technology will be used by local farmers in the country.^90^

Existing public trust models elucidate the complexity of the “trust” concept itself, as trust can be contextualized as shared values, or as trust in social actors or institutions across different attributes. A recent survey study of public opinion on sprayable RNAi-based pesticide in Italy has identified that trust in science and scientific literacy as key driving factors toward technological acceptance.^91^ They found that public skepticism toward RNAi technology should be addressed in a more holistic approach, by educational campaigns, building public trust, implenting evidence-based communication and ensuring transparency in the regulatory mechanism.^91^ Tardin-coelho concluded that building trust is the first step to initiate chances for transparent communication and public engagement on the RNAi topic. Furthermore, recognizing gaps in scientific knowledge as well as avoiding conflicts of interests among stakeholders could avoid mistrust on the use of RNAi-based pesticide.^44^

Exploring newer models of governance framework that contribute toward trust-building is crucial to preparing for emerging biotechnologies. The inclusion of public engagement and stakeholder outreach in the governance process is deemed necessary as these will provide inputs on ethical, social and cultural values that go beyond technical evaluation and expert opinions.^52^ Therefore, anticipation and reflection of possible social, economic or political impacts from an innovation will support the introduction of RNAi technology that aligns with societal values and needs, ultimately avoiding from conflicts and societal resistance of multi-stakeholders.^90^

Alternative governance approaches such as technological assessment and Ethical, Legal, Social Implications (ELSI) involve evaluation of technological risks and reflections on ethical and societal concerns of a technological development.^92^ While these governance models help to understand relevant concerns associated with a technology and protect society against undesirable consequences, they however failed to ensure that the introduction of a technology aligns with societal needs and values. In this context, the Responsible Research Innovation (RRI) framework has been proposed to promote opportunities to introduce a technology that is more responsive to societal needs.

RRI framework addresses a set of questions related to the complex, value-laden issues related to traditional regulatory requirements, bioethics and risk mitigation issues of biotechnology.^54,93,94^ RRI is defined as “an interactive process by which societal actors and innovators become mutually responsive to each other with a view on the (ethical) acceptability, sustainability and societal desirability of the innovation process and its marketable products in order to allow a proper embedding of scientific and technological advances in our society.”^95^ Underpinning the RRI framework is the consistent, ongoing involvement of a broad range of actors and stakeholders, who will reflect, anticipate and respond to risk governance challenges for biotechnology.^96^ The over-arching objective of RRI is to produce ethically acceptable and societally legitimate innovations fulfil specific public needs, increase the likelihood to obtain a social license to operate, which potentially could help to build trust in the technology and relevant actors.^13,97^

The RRI framework has four core dimensions of which each of the key tenets has unique concept and objectives, as well as techniques that can be applied.^54,96,98^ The key tenets are: i. *anticipation*: to describe and analyze expected or unexpected social, ethical, economic, political or environmental impacts that might arise due to scientific advances; ii. *inclusion*: to have dialogs with stakeholders and collectively discuss, construct and decide an optimum path for innovation, iii. *reflexivity*: to reflect on the underlying purposes, motivations and potential impacts, either on science and moral responsibilities; iv. *responsiveness*: to consider how the innovation process can respond to improve anticipation, reflexivity and inclusion.

Within this context, engaging with stakeholders to predict potential benefits and risks from the use of sprayable RNAi-based pesticides helps achieve the goal of responsible RNAi technology development under the pillar of “anticipation.” Under the same tenet, there should be a standard that a portion of public funding for RNAi-related research project should be allocated to investigate the environmental, social, legal, and ethical consequences of the technology.

To achieve the second pillar of RRI, “inclusion,” it would be desirable to organize dialogs with stakeholders especially representatives from farmer associations and consumer groups who will be the end-user of sprayable RNAi-based pesticides and collectively discuss and design a desirable innovation path in the early phase of the research and development process. In addition, engaging with social scientists, health and environmental risk analysts, ethicists, environmental advocacy groups should be considered to anticipate wider scientific, social, economic or political impacts of the technology and thus, allowing the technological developer to responsibly adjust the innovation process to maximize benefits and avoid potential harms.

Thirdly, the principle of “reflexivity” would require a deep understanding and reflection of intended and unintended impacts that could arise from the innovation process and the influence of such impacts on future societal issues. This notion manifests that relevant actors in the research and development should identify and evaluate their own practices and values to ensure the innovation process is conducted responsibly and ethically, and meet societal needs. For example, ascertaining the focus of an engagement should be a pre-requisite before engagements are made to allow participants to contest and raise societal challenges associated with the technology. The selection of participants is crucial as they should be speaking based on their knowledge, not because of neutrality and capability to represent certain interests.^99^

The final key characteristic of RRI, which is “responsiveness,” could be achieved by developing understanding on the relationship between communities within the agricultural sector and society as well as evaluating co-existing and competing visions for the future of agriculture. The purpose is to ensure responsiveness to diverse public stakeholder needs and perspectives and incorporate these multiple visions when charting the innovation path of the RNAi technology.

The past GM controversies have taught us a very important lesson about emerging biotechnological governance, which the framework should not focus only on risk governance but ideally should consider the societal element and moral responsibilities for innovation. Hence, the RRI governance model has been put forth to accommodate the need for an inclusive and democratic governance framework that put public interest as a priority. Inclusion of early engagement with the public and end users, as well as establishing collaboration guided by evidence-informed policy for RNAi-based pesticides is seen as a way forward to build and sustain trust in the technology.^13^ Yet, the vision of Responsible Research and Innovation (RRI) are difficult to implement because of many factors, such as funding constraints, regulatory challenges, institutional limitations, and potential public opposition.^100,101^ Ignoring these concerns may risk alienating those on the front lines of research and innovation, leading them to merely give nominal acknowledgment to RRI principles rather than genuinely adopting them. As an initial measure, providing incentives such as national funding policies and programs could be a useful approach, and compensation to RRI-practices researchers, technological developers and other institutions practicing RRI principles for possible delays in research development and capacity building for RRIs.^100^

## 5. Conclusion

In conclusion, as RNAi technology continues to evolve and discourses about ELSI concerns associated with the technology, the challenge of fostering public trust and increasing public acceptance remains critical. This review highlights that traditional risk-based governance approaches fall short of addressing public skepticism, as they often overlook societal and ethical dimensions. For the RNAi technology to be better accepted by the society, there is a need for a governance model that is not only scientifically robust but also ethically grounded, legally transparent, and socially inclusive. Central to this effort is the cultivation of trust, both in institutions and in the processes used to evaluate and implement RNAi-based technologies. The Responsible Research and Innovation (RRI) framework, with its emphasis on inclusivity, transparency, and stakeholder engagement, offers a promising pathway to enhance trust in biotechnology governance. By integrating ethical, social, and cultural values into the innovation process, RRI provides a more comprehensive governance approach that aligns with public expectations and mitigates distrust in emerging biotechnologies.
